# Women’s Insights on Extended Adjuvant Endocrine Therapy for Breast Cancer: Qualitative Online Forums Study

**DOI:** 10.2196/82016

**Published:** 2025-12-09

**Authors:** Géraldine Cazorla, Lorène Seguin, Magali Provansal, Sandrine De Montgolfier, Julien Mancini

**Affiliations:** 1 Aix Marseille Univ, Inserm, IRD, SESSTIM, Sciences Economiques & Sociales de la Santé & Traitement de l’Information Médicale, ISSPAM, Equipe CaLIPSo Labellisée Ligue Contre le Cancer 2024, Institut Paoli-Calmettes, 232 Bd Ste Marguerite, Marseille, 13009, France, 33 4 91 32 47 73; 2 Department of Oncology, Institut Paoli-Calmettes, Marseille, France; 3 Université Paris Est Créteil, Créteil, France; 4 APHM, Hôpital Sainte-Marguerite, BioSTIC, Biostatistique et Technologies de l’Information et de la Communication, Marseille, France

**Keywords:** breast cancer, endocrine therapy, online forum, netnography, decision-making

## Abstract

**Background:**

In France, breast cancer is the most commonly diagnosed cancer and the leading cause of cancer-related death among women. For around one-third of women with hormone receptor–positive breast cancer, extending adjuvant endocrine therapy (AET) beyond the initial 5 years is now recommended to reduce the risk of recurrence and mortality. While weighing benefits against potential side effects is essential, little is known about how women seek information about extended AET or how they experience this extension.

**Objective:**

This study aimed to explore, through online forums, women’s unmet information needs, the factors influencing their decision-making around extended AET, and the needs and expectations they share throughout the treatment journey.

**Methods:**

A qualitative content analysis was conducted using data from 5 French online forums over a 10-year period. We used a nonparticipant observational approach to identify relevant posts and gain a deeper understanding of forum dynamics related to extended AET. Data were collected in March 2025. After applying inclusion and exclusion criteria, 188 messages posted by 105 women were selected. A netnographic approach, derived from ethnography and suited to exploratory research, was used in conjunction with Braun and Clarke’s 6-phase framework for thematic analysis, integrating both inductive (narrative-based) and deductive (literature-informed) approaches.

**Results:**

Our findings showed that women turned to forums to seek information about extended AET from both medical and nonexpert sources. However, they often reported difficulties in interpreting complex or inconsistent information. Understanding and acceptance of treatment extension were influenced by the timing and manner of information disclosure. Three decision-making patterns were identified: (1) acceptance, (2) hesitation, and (3) refusal, which could shift over time, shaped by factors such as fear of recurrence, side effect experience and management, trust in treatment effectiveness, interactions with health care professionals, and life changes. Some women on extended AET shared resilient and encouraging experiences, providing reassurance and practical strategies for coping with side effects.

**Conclusions:**

Online forums offer valuable insights into women’s experiences, unmet information needs, uncertainties, and coping strategies surrounding extended AET, contributing to addressing a gap in knowledge on how women perceive and manage long-term treatment. Providing clear, accessible information and tailored communication tools, especially on benefit-risk balance and side effects management, at the time of prescription and at key points along the care pathway, may support informed decision-making, improve treatment adherence, maintain quality of life, and offer reassurance. With growing numbers of women in long-term treatment, enhanced coordination between primary care and hospital services, including follow-up by trained general practitioners and gynecologists in collaboration with cancer centers, may help ensure continuity of care and comprehensive support.

## Introduction

Breast cancer is the most commonly diagnosed cancer and the leading cause of cancer-related death among women globally [1]. In France, it accounted for more than 61,000 new cases reported in 2023 and remains the primary cause of cancer mortality in women with approximately 12,000 deaths recorded in 2018 [2,3].

Adjuvant endocrine therapy (AET) is a cornerstone treatment for early-stage hormone receptor–positive (ER+) breast cancer, which accounts for approximately 75% of cases [4]. Following surgery, radiotherapy, and when necessary, other adjuvant therapies, AET typically involves daily oral antiestrogens: tamoxifen (TAM) for premenopausal women and aromatase inhibitors (AIs) for postmenopausal women. The effectiveness of AET in reducing the risk of recurrence and breast cancer mortality is well established [5,6]. Despite these benefits, adherence to AET remains suboptimal. A systematic review of reviews [7] identified key determinants of nonadherence, including patient-related factors such as lower perceived necessity of AET and treatment concerns, health care provider (HCP) interactions, and socioeconomic factors. Evidence relating to medication- and condition-related factors is mixed, although side effects may play a relevant role.

For more than 3 decades, a 5-year course of AET has been the standard of follow-up care [5,8]. However, the risk of recurrence persists lifelong, particularly in patients with higher tumor grade, larger tumor size, and lymph node involvement [9,10]. For approximately one-third of patients, extending AET for 7-10 years is now recommended to reduce the risk of recurrence and death [11,12], with an absolute risk reduction of 2%‐5% at 10 years [13]. The optimal duration of extended AET remains uncertain, though [14,15]. A recent review suggests that extending AI treatment beyond 7 years provides only marginal benefits in disease-free and overall survival [16]. Moreover, a meta-analysis found no clear evidence of a benefit on overall survival with extended AET [17].

Extended AET carries treatment-specific risks, including increased risks of endometrial cancer and thromboembolic disease with TAM and higher risks of cardiotoxicity, bone fractures, and osteoporosis with AIs [18-20]. These adverse side effects, alongside those commonly reported during the initial 5 years, such as weight gain, hot flushes, sexual dysfunction, and depression [12,16,21], significantly impact quality of life. Consequently, decision-making around extended AET involves carefully balancing the potential absolute benefits of prolonged treatment against the risks and impact of side effects on women’s quality of life. This decision should result from a collaborative process between patient and clinician [4,11,22], grounded in medical ethics [23]. However, physicians often prioritize recurrence prevention over fully considering patients’ quality of life, sometimes downplaying the inherent uncertainties of offering a preventive therapeutic approach [24].

Women treated with a 5-year AET regimen have widely reported a range of challenges, including feeling either insufficiently informed at treatment initiation [25,26] or overwhelmed by complex and difficult-to-assimilate information [27]. Poor preparation for the severity of side effects—sometimes underestimated by HCPs—has been consistently documented [28]. Moreover, feeling insufficiently involved in decisions about their care has led women to seek information and support outside the medical setting, notably on the internet [26].

In the French context, a follow-up care pathway for patients with breast cancer may be provided by general practitioners (GPs) in primary care, in coordination with the hospital-based treatment team. Clinical follow-up is recommended every 3-6 months during the first 5 years and then annually, in accordance with the recommendation of the *Institut National du Cancer* (French National Cancer Institute) [29] and the *Haute Autorité de Santé* (National Authority for Health) [30]. Effective two-way exchange of information between GPs and cancer centers is considered essential [29]. However, qualitative studies conducted in France have highlighted that many women feel abandoned at the end of their institutional treatment and express a need for support and strategies to manage daily life after cancer while maintaining adherence to AET [31,32]. In particular, a perceived lack of information from HCP about AET and the management of early and late side effects may prompt some of them to turn to the internet and online discussion forums [31-33].

Over the past 2 decades, the digital revolution has reshaped the patient’s role, paving the way for the e-patient movement [34] and transforming the status of patients. The internet and social media have become indispensable sources of information for patients seeking medical advice, support, and emotional expression [35-37], while exponentially increasing the production and the dissemination of experiential knowledge through blogs, forums, and virtual communities [38].

In the context of extended AET, women are also likely to turn to online forums for information and peer support. A UK-based study using data from a breast cancer forum explored women’s decision-making regarding persistence with extended AET for up to 10 years [39]. Building on this, we investigated how this issue is experienced in the French context, through an exploration of 5 French online discussion forums, whether breast cancer–specific or not. While the UK study focuses on continuation up to 10 years, our objective was to take a broader view of how women perceive and experience any extension of AET beyond the initial 5 years. Specifically, we sought to identify potential unmet information needs, the main factors influencing decision-making with AET as well as how women express their needs and expectations regarding the overall experience of extended AET.

## Methods

### Study Design

This qualitative content analysis is based on a nonparticipant observational study of 5 French online discussion forums over a 10-year period (2015‐2025). It represents the first phase of a broader project, titled HORMONO+, examining the information needs, expectations, and lived experiences of women undergoing extended AET, with the aim of identifying strategies to improve follow-up care. In this initial phase, we adopted a netnographic approach, a qualitative method pioneered by Kozinets [40] derived from ethnography, which is particularly well suited for exploratory research as it allows nonintrusive observation of users’ online social interactions and experiences, offering rich insights into shared information and a deeper understanding of behaviors in digital contexts [41]. We used the SRQR (Standards for Reporting Qualitative Research) checklist to ensure completeness and transparency in reporting our qualitative findings.

In February 2025, we conducted a search on Google to identify online discussion threads related to extended AET, which led to the identification of 5 French-speaking, France-based forums. In line with current best ethical practices in internet forum–based research, we chose not to disclose the names of the forums [42-44] and, instead, assigned each forum a letter of the alphabet. These forums were hosted on general health-related websites (forum A), platforms dedicated to chronic diseases and cancer (forums B and C), or were specifically focused on breast cancer (forums D and E).

**Figure 1.**
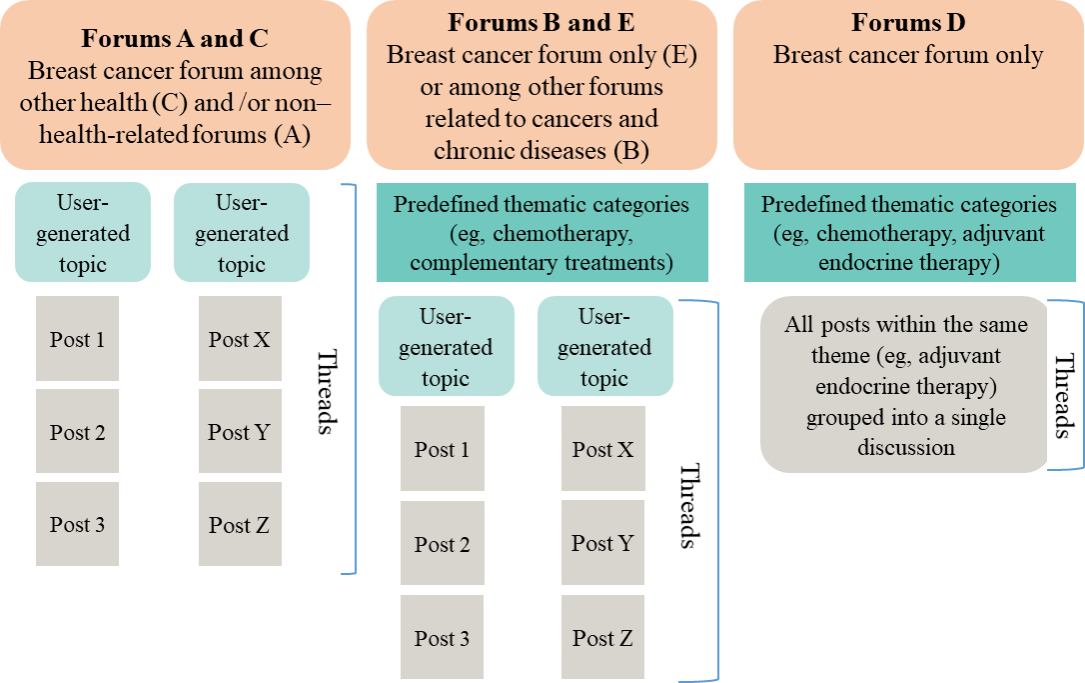
Structure of 5 French online forums studied in relation to breast cancer and extended adjuvant endocrine therapy as part of the qualitative netnographic analysis conducted within the HORMONO+ research program.

The structure of discussion threads varied across forums. In some (A and C), users could freely create discussions and post messages on a wide range of breast cancer–related topics without strict categorization. Other forums (B, D, and E) featured a more organized layout, especially forums D and E, where discussions were divided into predefined thematic categories (eg, chemotherapy, radiotherapy, and complementary treatments). In forum E, each thematic category contained multiple discussion threads, each addressing a specific topic. In forum D, however, all messages related to a given theme (eg, hormone therapy) were grouped into a single, often very large, discussion thread which could contain thousands of posts. For simplicity, we referred to each thematic category as a “discussion thread” (Figure 1).

Some forums were moderated by a qualified health care professional (C) or by nonprofessional moderators (B), some of whom had personal experience with breast cancer (D and E). Moderator involvement varied from proactive engagement to retrospective oversight. Forum guidelines included recommendations regarding personal responsibility, confidentiality, and security (eg, avoiding disclosure of personal information), as all messages were publicly accessible, except in forum B, where registration was required to access the full content of all messages.

Although some websites reported high traffic (more than 4 million unique visitors per month in 2025 for the website hosting forum A) and gathered thousands of members (more than 3000 for forum B), traffic volume was not the primary selection criterion. Instead, the decision to include different types of forums was driven by the aim to ensure greater diversity and capture a variety of user profiles and experiences. While users’ geographical locations were not always specified, the majority appeared to be based in France, although posts from French women living abroad were also identified.

### Data Collection

**Figure 2.**
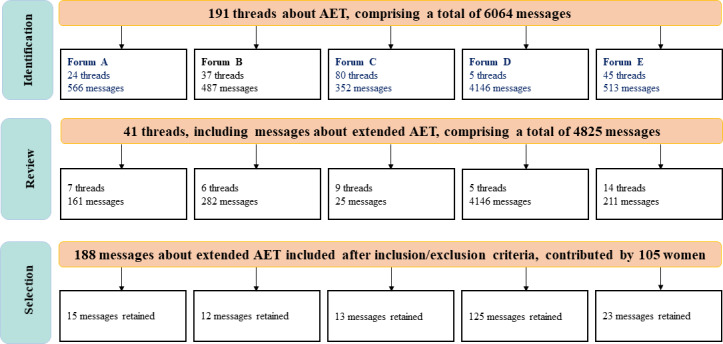
Flowchart for the process of identification, review, and selection of messages related to extended AET in women with breast cancer, from 5 French forums in March 2025, as part of the qualitative netnographic analysis conducted within the HORMONO+ research program. AET: adjuvant endocrine therapy.

For each forum studied, we first conducted nonparticipant observations to better understand forum dynamics and identify discussion threads relevant to our research topic. Data were collected in March 2025, from discussion threads in forums A-E addressing AET, by either directly mentioning the term or referring to specific treatments (TAM, anastrozole, and letrozole) using either their generic or brand names, over a period spanning a decade (2015-2025). A total of 191 discussion threads were identified based on these keywords, comprising 6064 messages during the specified period. Forty-one threads specifically discussing extended AET were retained (Figure 2).

We then included posts from women, either pre- or postmenopausal, undergoing AET for ER+ breast cancer, with TAM or an AI, meeting the following inclusion criteria: (1) having received AET for at least 5 years, (2) meeting medical criteria for extending AET beyond 5 years, (3) eager to continue AET beyond 5 years even in the absence of medical indication, and (4) discussing AET extension regardless of treatment duration. Posts from family members discussing treatment extension for a relative were also included. We excluded posts from patients with metastatic breast cancer and men diagnosed with breast cancer. A total of 188 relevant messages posted by 105 women were included in the analysis.

All relevant posts were copied and pasted into an Excel spreadsheet and organized thematically to provide an overview of key discussion areas. For each message, the forum identifier (A, B, C, D, or E), the thread title, the message URL, and the publication date were systematically recorded. All relevant posts were copied and thematically organized to provide an overview of key discussion areas with the forum identifiers (A-E), the thread title, URL, and publication date.

### Data Analysis

Some elements of the thematic analysis derived from netnography [41], including the identification and selection of online communities, data collection, analysis, and interpretation, were integrated with Braun and Clarke’s approach [45] whose six steps were implemented as follows: (1) identification of discussion threads, reading of posts, and note-taking (GC); (2) development of initial codes using both inductive (narrative-based) and deductive (literature-informed) approaches on 20% of the dataset (GC and SDM); (3) generation of potential themes and subthemes through team discussion (GC, SDM, and JM); (4) refinement and revision of themes and subthemes (GC); (5) consensus on the final thematic framework (GC, SDM, and JM); and (6) independent coding and analysis by 1 researcher (GC).

### Ethical Considerations

The research project titled “HORMONO+” was approved by the ethics committee of Aix-Marseille University at its meeting on February 27, 2025 (IRB00014113, reference no.: 2025-02-27-012). No informed consent was sought, as the messages posted by forum users were publicly available and there was no direct interaction with users in this nonparticipant observational study. To preserve anonymity, forum pseudonyms were replaced with generic female first names, and any identifying details were removed. Forum posts were reworded for publication to maintain their meaning while preventing traceability to individual users. No financial compensation was provided.

## Results

**Figure 3.**
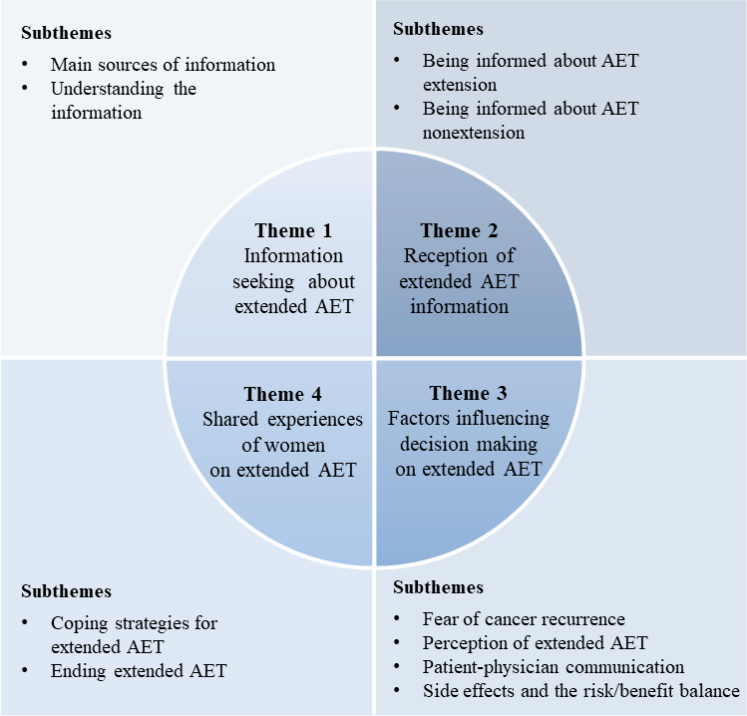
Thematic framework of themes and subthemes related to extended AET in women with breast cancer, derived from the qualitative netnographic analysis of 5 French online discussion forums. AET: adjuvant endocrine therapy.

Through our analysis, we identified 4 major themes around which women’s discussions on extended AET revolve (Figure 3).

### Theme 1: Information Seeking About Extended AET

#### Main Sources of Information

Analysis of discussions shows that women actually seek information to better understand the clinical and scientific rationale for extending AET beyond 5 years. Their knowledge-building process draws on both biomedical sources and nonexpert or experiential insights.

#### Biomedical Knowledge

As early as 2015, posts discussing extended AET can already be found, likely in connection with the 2012 ATLAS study [18], which is also mentioned by a moderator. Throughout the observed period, women continue to seek information in light of evolving international and national guidelines. When recounting how they first heard about the possibility of extending AET, they frequently cite scientific sources, referring to them as “studies,” “research,” “protocols,” “statistics,” and so forth.

*I attended a workshop on hormonal therapy, and indeed, the idea of extending this treatment to 10 years is currently being studied. It’s still under investigation*.

[Carole, exact duration of AET not specified, 2015, Forum D]

Some women occasionally share the sources they rely on (such as the name of a conference, a study link, or a webinar), thereby fostering interactions between users and facilitating access to evidence-based information.

*Thank you for this interesting link* [to a webinar organized by a leading institute]. *It’s very well done, [...] but there still aren’t no long-term studies on the* [optimal] *duration of hormone therapy...*

[Marie, 5 years on AET, 2023, Forum D]

Links to papers published on general health websites are also shared.

#### Nonexpert and Experiential Knowledge

Some women, despite receiving information directly from their HCPs—primarily oncologists—seek opinions from other forum users:

*My oncologist says we are behind the US, and the new protocol is 10 years. What do you think about this?*

[Martine, 7 years on AET, 2019, Forum B]

In some cases, the trust placed in peer-shared information may match or even surpass that placed in HCPs, with both forms of knowledge complementing each other. Users commonly call on community members to share advice and information or insights based on their understanding and personal experience with extended AET. One user draws on both scientific knowledge (without citing sources) and personal experience to explain the rationale for extending treatment:

*It’s well established that extending treatment to ten years provides a benefit, but not beyond that, as it may lead to additional negative effects [...*] *It’s important to monitor the uterus carefully throughout treatment. Personally, I was able to take Tamoxifen for seven years-the first 5 years without any issue-then my endometrium began to thicken*.

[Isabelle, completed 7 years on AET, 2018, Forum D]

Forum users are sometimes consulted about specific clinical situations, often due to uncertainty arising from information perceived as too vague. While most requests seek general advice, they can occasionally place women in an ambiguous position, especially when their “medical opinion” is sought or they are directly asked “what to do” when facing a treatment decision.

*In* [month], *it’ll be 5 years on* [TAM]. *I’m torn - should I stop or continue for another five? They’ve mentioned studies recommending continuation...I’m feeling a bit lost and need some advice*.

[Joan, nearly 5 years on AET, 2018, Forum D]

Several contributors, however, urge others to prioritize the guidance of HCPs who, as one woman notes, “have studied for years and presumably know the subject well” (Louise, 5 years on AET, 2018, Forum D). Others highlight the limits of anecdotal evidence, advising women against drawing conclusions from individual cases of recurrence or remission:

*Making your decision based on examples of recurrence—or not—is just forming an opinion. But doctors don’t prescribe based on opinions; they rely on evidence from randomized trials*.

[Beatrice, completed 5 years on AET, 2019, Forum D]

#### Understanding Information on Extended AET

Regardless of its source, information about treatment extension is sometimes poorly understood. Several women report difficulties grasping the content, describing studies as “not all consistent,” with “contradictory statistics,” and noting that doctors are “divided” or “disagree.” They also mention results that seem to carry “no certainty,” or appear “unreliable.”

Such ambiguities can result in misinterpretation of medical information and lead to extrapolations. For example, in forum C, when a woman expresses surprise at her extended treatment protocol, another suggests that it might be related to lymph node involvement, while a third says that it may not be, as her own lymph nodes were negative. Beyond medical speculation, the complexity of navigating information leads some users to develop controversial theories, which may foster a sense of suspicion. For instance, one woman interprets the extension of treatment as an act of state interference.

*My oncologist first said 5 years, and now it’s at least 7 years. She said the government is getting involved...I got the feeling she’s not doing exactly what she wants, which I find unacceptable*.

[Tiffany, initially prescribed 5 years on AET, extension recommended to 7 years, 2025, Forum C]

Some women question the necessity of extended AET (“Is it necessary in all cases?”), perceiving this recommendation, which “will cost more,” as possibly driven by the pharmaceutical industry interests, which “benefit greatly from this ‘cash cow’,” while “patients’ interests come far behind financial ones.”

*Today, my oncologist told me that recently, the treatment has been extended from 5 years to 7 or even 10 years...And I’m questioning the* [influence of] *pharmaceutical lobbies*.

[Adriana, 5 years on AET, extension recommended to 7-10 years, 2017, Forum D]

### Theme 2: Reception of Information on Extended AET

Women’s reactions to extended AET vary depending on when they received the information. Some expressed surprise when the duration was revised midcourse, while others were informed from the outset that it could last up to 10 years and had already factored in this possibility.

#### Experiencing a Revised Treatment Plan Midway

Women cite scientific arguments such as “new publications,” “a study,” or “a recent conference” to justify their clinician’s recommendation to extend a treatment initially planned for 5 years. Beyond the initial surprise, the disappointment caused by this change can be considerable.

*I thought I’d be done after 5 years, but now they’re saying it might be extended by another two! I’m feeling pretty disheartened and honestly quite fed up*.

[Jana, 5 years on AET 5 years, extension recommended to 7 years, 2015, Forum D]

For another woman, continuing beyond the originally prescribed 5 years is seen as unmanageable, since that point had been considered a clear end point.

*I held on because I believed it would end—I drew on all my mental and physical reserves, just to make it to that point*.

[Lana, 5 years on AET, extension recommended to 10 years, 2022, Forum D]

#### Experience When Extended AET Is Announced at Initiation

Some women report being informed “from the outset” that treatment would last “at least 10 years.” Phrases such as “I was told,” “they announced,” or “the oncologist said,” followed by mention of an extended duration, frequently appear in their accounts.

#### Reactions to Extended AET

Whether introduced at initiation or later, the recommendation for long-term treatment generally elicits 3 types of response: acceptance, hesitation, or refusal—each of which may evolve over time depending on individual circumstances and a range of factors explored in theme 3.

##### Acceptance

For some women, acceptance is sometimes tinged with resignation, revealing a tension between adherence to the medical protocol and the feeling of having no real alternative.

*My oncologist warned me in advance, giving me some hope that maybe they’d stop* [AET] *after 7 or 8 years, depending on my condition, but I’m not fooling myself. I know I’ll stick with it despite the side effects if it’s the “price” to pay to avoid a recurrence. It’s worth trying*.

[Jeanne, 3 years on AET, recommended for 10 years, 2016, Forum D]

For others, accepting extended AET appears to be a more proactive choice, supported by active research into validated information and the possibility of reevaluation based on clinical progression.

*was wondering whether to continue or stop...I was advised to watch* [name of webinar], *so I did. I’ve decided to continue for another 5 years as long as I don’t get severe osteoporosis. The explanations were clear, easy to follow, and above all, encouraging*.

[Sonia, 5 years on AET, 2023, Forum D]

One woman, while sharing the link to a paper, emphasizes the importance of taking an active role in the therapeutic decision-making process.

*I’ve just been told I’ll need 7 years of hormone therapy...not 5, as recent studies say it should be extended! I’m staying “reasonable” for now and, of course, I’ll do what’s proposed, but not without discussing it first!*

[Patricia, ≤1 year on AET, recommended for 7 years, 2015, Forum D]

##### Hesitation

The severity of side effects is the primary reason cited by women who hesitate to continue AET.

*My oncologist recommended another 5 years [...*] *I felt completely demoralized and don’t know what to do, I’m already struggling with so many side effects*.

[Adele, 5 years on AET, 2016, Forum D]

Hesitation may also stem from the dilemma of whether to continue an already extended AET given potential health risks. In such cases, some women may turn to peers for guidance in their decision-making, such as illustrated by a user experiencing endometrial complications:

*I feel like I have two options: either I stop taking* [TAM] *knowing that I won't have completed the 10 years my oncologist recommended, or I accept a new antiestrogen treatment [...] I'm really uncertain right now...I’m grateful to all forum members who will [...] share their experiences, to help me decide whether to continue or to free myself by stopping the hormone therapy*.

[Edith, 6 years on AET, 2019, forum D]

When faced with perceived indecisiveness from HCPs regarding therapeutic choices, some women also seek further insights from peers.

*I’m unsure...but when I see this study* [shared by a peer], *I feel I should continue...My oncologist is hesitant* [because of the risk of osteoporosis]*...he even said that if I stopped but stay well monitored, I could start again if needed to block everything...? Still, I have doubts*.

[Julia, 6 years on AET (ultimately continued for up to 9 years), 2016, Forum E]

Regardless of their actual influence, messages encouraging continuation are found across different forums. Some take on an almost prescriptive tone:

*If I were you, I wouldn’t hesitate—I’d continue*.

[Svetlana, exact duration of AET not specified, 2016, Forum E]

Others encourage putting the situation into perspective:

*As a mother, if my girl had chosen to stop hormone therapy, I would have urged her to continue. Hormone therapy is tough, but cancer is far worse*.

[Angel, mother of a woman on AET for 5 years, 2015, Forum A]

##### Refusal

The decision not to continue treatment is most commonly voiced by women who were initially told that they would undergo a 5-year protocol and had been anticipating its completion. In these cases, disabling side effects are the main reasons cited, although this does not necessarily make the decision any easier.

*I told my oncologist I didn’t want to continue, and he didn’t push me to carry on. Still, I feel a bit unsettled. Can anyone help me with this?*

[Adriana, 5 years on AET, 2017, Forum D]

However, the decision can shift over time as various factors come into play. Looking back over earlier posts, it is not uncommon to find women who, having initially rejected the idea, eventually chose to extend AET.

### Reactions to AET Nonextension

Among women on the forums who do not medically require AET extension beyond 5 years, stopping treatment brings ambivalent feelings. Joy and relief are mixed with a sense of unease, expressed in their discourse through phrases such as: “I’m walking without a safety net,” “I won’t feel protected anymore,” or “We’re never truly reassured or at peace” or even referring to their treatment as “an old friend.”

*I’m nearing the end of my* [...] *treatment, which is great news, but I’m also a little apprehensive. It feels liking saying goodbye to an old friend who wanted the best for me! Funny how you get used to it!*

[Olivia, completed 5 years on AET, 2015, Forum D]

Some women and their families report experiencing or observing physical and psychological symptoms following treatment discontinuation. Emotional distress is described with expressions such as “a fear that only leaves me when I sleep,” “completely shaken and destabilized,” and “sleep problems, anxiety.” A potential link between these feelings and the perceived loss of therapeutic protection deserves further investigation.

In addition to concerns about these symptoms, the distress caused by the lack of medical follow-up or guidance is also highlighted.

*Since stopping the treatment, her husband and the whole family have been very worried because she* [their daughter] *has become highly aggressive and very negative, which is affecting family life. Could there be a link between stopping hormonal treatment and these issues? We really don’t know who to turn to*.

[Aline, mother of a woman who completed 5 years on AET, 2024, Forum C]

Several women express a sense of regret at not being able to continue AET, varying in intensity, and sometimes even conveyed with a touch of humor.

*Last year, my oncologist recommended stopping, but I didn’t want to. He actually laughed a lot because it was the first time a patient asked to continue...So, I stopped, though reluctantly, because continuing made me feel reassured and today, everything is going well*.

[Lydia, completed 5 years on AET, 2024, Forum A]

Beneath the regret, deeper introspection sometimes emerges, focused on managing future regrets and perceived responsibilities.

*If I had been offered another 5 years, I would have signed up, because I know I’ll feel less protected...I don’t want to end up one day wondering “what if I had gone all the way?” and have to bear the responsibility if a relapse occurs*.

[Lea, completed 5 years on AET, 2015, Forum A]

Occasionally, the medical decision to limit treatment to 5 years sparks genuine confusion, accompanied by feelings of injustice and a sense of lost therapeutic opportunity. This is the sentiment expressed by Marie, who has made extending treatment her cause (Textbox 1).

Textbox 1. **Contextualized extracts of forum messages from Marie, “extending adjuvant endocrine therapy at all costs” (5 years on adjuvant endocrine therapy, 2023, forum D), as part of the qualitative netnographic analysis conducted within the HORMONO+ research program.**

Marie was prescribed a five-year course of AET. Early on, this active forum user expressed a desire to extend it beyond this period, despite medical advice suggesting it was unnecessary. Her “fear of recurrence” seemed to outweigh scientific arguments, as she stated: “*Statistics help doctors choose protocols; for me, they matter little because you never know which side of the statistic you’ll fall on*.” Drawing from her experience, she highlighted several cases of recurrence after AET cessation, even in patients considered low risk. Her use of quantifiers (how many women; too many) accentuates the sense of frequency: “*I know many ‘low-risk cancers’ that metastasized 10 years later*.”

While Marie advocates for extension as a personal decision, she acknowledges the need to weigh it against side effects, age, and risk perception. Some of her statements, however, may suggest that continuing treatment is the only viable option: “*Knowing that hormone therapy prevents any remaining cancer cells from growing, what will stop them from waking up and growing once treatment stops?*” The potential influence of such messages on vulnerable or uncertain users’ decision-making processes raises concerns. Finally, although Marie laments the “*lack of oncologist-patient dialogue*,” believing the decision should be mutual, she praises the support from her general practitioner and gynecologist, who agreed to prescribe treatment beyond five years.

### Theme 3: Factors Influencing Decision-Making on Extended AET

The decision to accept or refuse extended AET appears to be influenced by a combination of interrelated factors, including fear of recurrence, beliefs and attitudes toward AET, the patient-HCP relationship, and side effects.

#### Fear of Cancer Recurrence

Fear of recurrence frequently emerges as a central factor in the decision to commit to long-term treatment. This fear is expressed with varying degrees of intensity, ranging from a simple “fear” to “terror,” “anxiety,” “panic,” and “dread.” It also surfaces through vivid metaphors such as the image of “dormant little clones [that] can awaken and no longer face obstacles to their activity” once therapy ends.

#### Perception of Extended AET

The discourse surrounding extended AET frequently draws on the lexical field of protection. It is metaphorically described as a “shield,” a “safety net,” “crutches,” a “chance,” or a form of “security,” conveying a sense of reassurance. While some view it as “a guarantee of survival,” others adopt a more measured perspective, acknowledging that although it “significantly reduces the risk of distant recurrences,” it is “not necessarily a ‘full-coverage’ guarantee.”

*For me, hormone therapy is like car insurance: I pay [the high cost of side effects], without knowing if it will be of any use [prevent a recurrence or not]. Still, I’ve made my choice and keep paying even though at times I feel like suspending payments! The important thing is that everyone can choose in good conscience*.

[Alice, 3 years on AET (continued beyond 5 years), 2016, Forum D]

Trust in the effectiveness of the medication appears to be a critical factor influencing long-term persistence with AET:

*[...*] *yes, I have side effects [...*] *but it’s still manageable, so I’ve just decided to “sign up” for 3 more years because I firmly believe in its effectiveness*.

[Ines, 7 years on AET, 2022, Forum E]

This confidence is reflected in the use of affective and personified language to describe AET, with terms such as “little pill,” “best/old friend,” “companion,” “ally,” “sweet” or even comparisons to a “favorite dessert” used to characterize different molecules. For some women, strong belief in the effectiveness of a specific molecule can lead to a deep attachment, especially when side effects are manageable. In certain cases, this attachment is so strong that even when medical complications arise (such as endometrial inflammation or polyps) that would normally warrant discontinuation, they consider taking drastic steps to remain on that particular molecule.

*[...*] *tamoxifen causes uterine and endometrial cancers, but not ovarian cancer, right? If that’s the case, I’ll have my uterus removed, keep my ovaries, and continue tamoxifen for 10 years without added risks—offering greater protection, no extra concerns, no menopause, and thus, no major side effects impacting my quality of life. Is this a ridiculous idea, or does it make sense*?

[Agnès, 4 years on AET, 2019, Forum D]

Across various forums, the perception of the effectiveness of extended AET tends to be generally positive among women concerned, sometimes persisting even after discontinuation.

*Tamoxifen was the medication that saved me for several years, even after I stopped hormone therapy, especially since I took it continuously and very regularly for 10 years*.

[Elena, completed 10 years on AET, 2021, Forum B]

Regret over continuing treatment beyond the initial 5 years is rarely expressed, even among those who experience severe side effects. In contrast, skepticism regarding extended AET’s efficacy tends to be voiced by women who were hesitant from the outset and either declined to initiate AET or discontinued it early.

#### Patient-Physician Communication

A trusting relationship with HCPs, built on attentive listening, nonjudgmental attitudes, and clear, informative communication, appears to facilitate acceptance of extended treatment, particularly when patients feel involved in the decision-making process. Conversely, relationships perceived as hierarchical, coupled with inadequate communication, may engender doubt or resistance.

#### Shared Decision-Making

The feeling of full involvement in decision-making is expressed in various ways. For some women, it is shown by using the pronoun “we,” sometimes even in capital letters:

*After discussing it with my oncologist, I tried going without treatment for a few months. And since I’d already done 8 years, WE decided to stop. Just to be clear: this wasn’t an impulsive decision.*

[Claire, completed 8 years on AET, 2024, Forum A]

Expert patient Hannah (completed 15 years on AET, forum E) encourages forum users to actively engage in discussion with their HCPs: “try to understand their medical strategy” and “express their feelings and wishes” while also trusting their “personal intuition.” Others explain that although the decision to continue treatment was made together with their doctor, in the end, they decided on their own, sometimes following discussion and negotiation with HCPs:

*After 5 years of tamoxifen, with its ups and downs, I chose to continue. For how long, we’ll see?! My oncologist and gynecologist agreed, but the decision was mine*.

[Alice, 5 years on AET, 2018, Forum D]

*I had to push my doctors, some wanted me to stop after 5 years. I negotiated 2 more, then another 3* [years].

[Michaela, completed 10 years of AET, 2022, Forum D]

The role of HCPs can be pivotal in case of uncertainty, as shown by 2 posts from the same user 1 day apart. Initially overwhelmed by side effects and reluctant to extend treatment, she decided to continue after consulting her psychologist and began to regain hope.

#### Perceived Inadequate Communication and a Relationship Viewed as Hierarchical

Some women report feeling insufficiently informed. This is the case of Lana (Textbox 2), nearing the end of her 5 years on AET, who clearly expresses a need for information and documentation to make an informed decision.

Textbox 2. **Contextualized extracts of forum messages from Lana, “adjuvant endocrine therapy extension under condition” (5 years on adjuvant endocrine therapy, 2022, forum Forum D, as part of the qualitative netnographic analysis conducted within the HORMONO+ research program.**

As she approaches the end of her 5-year treatment plan, Lana is informed that extending it by another five years could be beneficial. However, she perceives this as more of a directive than a choice: “*My new oncologist (the previous one, who was great and non-judgmental, moved to private practice) is offering? imposing? an additional 5 years of aromatase inhibitor treatment...She tells me that a study shows a 10-year protocol is highly beneficial for my case. But without providing figures or explanations, and I can’t find any documentation to make an informed choice*.”

Feeling that the information provided by her clinician is inadequate, Lana turns to the forum, seeking “*more precise, comparative data with figures and stats*” to prepare for her upcoming discussions with her oncologist. Earlier in her treatment, Lana had already stressed the importance of being properly informed about side effects, particularly at a time she was questioning the continuation and overall benefit of AET: “*It seems to me that requesting the right information to make an informed decision and own it is a patient’s right, and that’s all I’m asking for*.” Faced with the prospect of extension, this need for clear information becomes more crucial than ever in her decision-making process, alongside quality-of-life considerations: “*I will indeed seek clear explanations because this infantilizing medical approach is intolerable to me. And of course, I’m tempted to try [to continue] but if it means enduring sleepless nights or pain again, I'll stop*.”

The perception of an asymmetrical relationship with HCPs is reflected in language-evoking obedience (“I comply” and “swallowing the pill without thinking too much though I feel fed up”). This obedience is sometimes expressed ironically, using terms such as “good little soldiers.” In certain cases, medical recommendations are perceived as leaving little or no room for personal deliberation, leading some women to feel sidelined in the decision-making process.

*Do I really have a choice? They told me it’s ten years of hormone therapy. So, what am I supposed to pick, the hammer or the anvil*?

[Caroline, 5 years on AET, 2022, Forum D]

Several women describe feeling unheard, particularly when reporting side effects “not mentioned as usual by doctors.”

*They really do take us for fools. It’s a relief to talk here, because I see I’m not the only one with side effects—side effects that some doctors won’t even acknowledge, though they’re clearly listed in the leaflet*.

[Morgane, 3 years on AET in 2017, (completed 8 years in 2023), Forum D]

Forums thus offer peer support to share experiences and concerns that may be perceived as overlooked by HCPs, potentially fostering greater autonomy in decision-making. This empowerment is echoed in terms such as “sisters in struggle” or “warriors” or expressions of agency such as “I decided,” “I refused,” and “the decision is ours.” By providing a space for such affirmations, forums seem to help counterbalance the asymmetry sometimes observed in patient-HCP relationships. This dynamic is reinforced by messages urging women to seek clear information, question medical decisions, or even change practitioners.

#### Side Effects and the Benefit-Risk Balance

Side effects reported by women vary in type and perceived severity. The most commonly cited include fatigue, hot flashes, joint and muscle pain, weight gain, insomnia, and loss of libido. Less often mentioned are digestive issues, mood disturbances, hair loss, and perceived cognitive decline. Some women link these symptoms to AET or treatment changes, while others suggest that they may be related to menopause. The perceived severity of side effects varies, from “very manageable” to “hard to bear,” and seems to play an important role in women’s decisions about continuing AET. For one woman, fear of recurrence and the desire to celebrate her grandson’s 20th birthday outweighed the burden of side effects, prompting her to continue treatment.

*I accept enduring it because it increases my chances of avoiding a recurrence. I keep* [my oncologist] *informed about all these side effects; he tries to help me*.

[Fanny, 6 years on AET, 2017, Forum D]

In other cases, the decision not to extend treatment reflects a personal assessment of clinical, statistical, and individual factors, with or without involvement of the HCP.

*[...*] *my oncologist told me it’s up to you to decide if you want to continue, if it reassures you!!! Surprised, I asked for more details*. *[...*] *So, I stopped 4 months ago*.

[Marina, completed 5 years on AET, 2017, Forum D]

*I reviewed the statistics regarding my cancer, all the issues with hormone therapy, and my age* [>70 years]*...I didn’t mention it to my oncologist; he wants to extend hormone therapy for another 5 years. I don't want a lecture. This decision* [to stop] *is personal. If I were 20 years younger, of course, I would continue the treatment*.

[Charlotte, 5 years on AET, 2022, Forum D]

A desire for pregnancy is occasionally cited as a reason for not extending treatment beyond the initial 5 years or for limiting its duration. One woman, for example, agreed to continue for up to 7 years but no further, in order to preserve her chances of conceiving before the age of 40 years. To support the deliberation around AET extension, some contributors occasionally provide a list of specific questions for community members to consider, addressing issues such as the risk of recurrence without treatment, the potential benefits of continuing therapy, the possible influence of comorbid conditions on side effects, psychological factors, and possible therapeutic alternatives. Fellow forum members are encouraged to reflect on these dimensions, which may influence or potentially bias their decision-making process.

### Theme 4: Shared Experiences of Women on Extended AET

#### Overview

Despite the varying severity of side effects, encouraging accounts of women on extended AET are relatively common on online forums. These contributions, often marked by resilience, urge others to adopt a balanced perspective. In addition to offering reassurance and inspiration, these women also share practical tips and personal strategies to manage side effects. Some women encourage others to approach what they read and post on forums with a critical—and at times self-critical—eye, warning against the magnifying effect of social media.

*It’s true that we tend to post messages when something goes wrong*.

[Catherine, 4 years on AET in 2015 (extended to 7 years), 2015, Forum D]

Others go further, stating that they “never really questioned the side effects” or considered stopping treatment, viewing it as essential “for staying alive.” However, such calls to downplay concerns can sometimes be poorly received, as illustrated by this message which was perceived as judgmental and sparked strong reactions within the community:

*I don’t obsess over the pills, remission, recurrence risks or survival rates. I NEVER think about any of that! For heaven’s sake, just live normally! Stop stressing over whether you’ll relapse or not...*

[Alba, 6 years on AET, 2018, Forum D]

#### Coping Strategies for Extended AET

Several women attribute their persistence to an early acceptance that “the benefits are greater in the long term, provided the side effects remain tolerable,” along with an understanding of the importance of treatment. Others describe using psychological coping strategies such as positive mental imagery and accommodative coping to better manage adverse effects:

*I picture the medication doing its job inside my body and tell myself: you must, you must keep going...*

[Samia, 13 years on AET, 2024, Forum C]

Faced with a broad range of side effects, women report using what one participant calls “a whole arsenal of tactics to ease them.” These encompass both pharmacological treatment (painkillers, antidepressants, and sleep aids) and nonpharmacological approaches (physiotherapy, osteopathy, acupuncture, hypnosis, and auriculotherapy). Complementary and alternative therapies such as homeopathy, herbal teas, cannabidiol, and dietary supplements are also cited. Physical activity adapted to individual health status, including walking, hiking, gentle exercise, swimming, cycling, running, or yoga, is frequently mentioned, as is meditation. However, some women stress that there is no miracle cure and advocate for personal coping strategies over time, according to one’s needs and capacity for resilience:

*Some days I feel like hitting the first person I see [...*] *I don’t believe there’s a real cure, except self-control, meditation...maybe some magnesium...and just trying to calm myself down*.

[Emma, 8 years on AET, 2018, Forum D]

#### Ending Extended AET

Finally, a similar ambivalence is observed both in women who complete extended AET and those who stop at 5 years, as initially prescribed. Relief and a sense of returning to normal coexist with worry, feelings of lost protection, and lingering doubts (“what if stopping was a mistake*...* what if I should have continued*...*”).

*My oncologist says I don’t need to go on to the full 10 years. I’m torn between thinking “great, I’ll finally feel free again” and “oh no, I’m without a parachute.”*

[Florence, completed 7 years on AET, 2019, Forum D]

The tension between relief and apprehension suggests the importance of providing clear information and reassurance to better prepare women for the end of treatment.

## Discussion

### Principal Findings

Our study provides novel, comprehensive insights into how women in France engage with online forums to share information, expectations, and experiences regarding extended AET. While extensive quantitative and qualitative research has examined determinants of adherence during the first 5 years of treatment [7], qualitative studies focusing on women undergoing extended AET remain limited. This study addresses this gap by exploring women’s information needs regarding extended AET, the factors influencing their decision-making, and how they share experiences and manage side effects over time, an issue of growing importance as an increasing number of women are now prescribed extended therapy, highlighting the value of online forums as a rich source of information for a deeper understanding of these dynamics.

Our findings suggest a proactive approach to gathering knowledge from both biomedical and nonexpert sources. However, difficulties in interpreting information perceived as complex or inconsistent may lead to misconceptions, underscoring the critical need for clear, reliable, and accessible information, especially regarding the risk-benefit balance of extending AET.

Women’s reception of information about extended AET appeared closely linked to the timing of its disclosure. Those who were initially prescribed a 5-year protocol occasionally expressed surprise or disappointment, while women informed from the outset about potential treatment extension seemed more likely to have integrated this possibility. Regardless of when the information was conveyed, 3 patterns of decision-making typically emerged among forum participants: acceptance, hesitation, or refusal. Interestingly, for women not medically required to continue AET beyond 5 years, discontinuation was sometimes accompanied by ambivalent feelings—relief mixed with a sense of lost protection. Some even reported feelings of injustice or the perception of being denied a potential therapeutic opportunity.

The time frame of our data collection (2015‐2025) showed that reasons for continuing, discontinuing, or being undecided about extended AET may evolve over time, influenced by individual circumstances and various factors. These include fear of cancer recurrence and prioritizing survival over immediate quality of life, experience and management of side effects, and trust in medication effectiveness. These factors have also been identified in previous research on persistence with AET over both a 10-year period [39,46] and within the standard 5-year treatment regimen [25]. Regarding side effects, our data indicate that their presence alone did not necessarily result in treatment discontinuation [39]. Instead, this seemed linked to women’s self-efficacy to manage symptoms [47], along with the use of both pharmacological interventions (eg, antidepressants) and nonpharmacological approaches (acupuncture, yoga, meditation, and physical activity), some of which are supported by the best available evidence [48].

The feeling of being insufficiently informed by HCPs about the benefits and risks of extended AET, especially regarding side effects, or receiving conflicting information from physicians was another important factor influencing both decision-making and the overall experience of extended AET. This aligns with findings from research on extended AET [39,46] and the standard 5-year regimen [7,25]. As shown in our study, even among women who have been on treatment for several years, the perceived lack of clear, specific, and personalized information about extended AET may lead some to seek information independently [49] or turn to online discussion forums for guidance [26,39], including in their decision-making process. Exploring data from online health discussions is thus particularly valuable for capturing patient perspectives —especially as patients with breast cancer tend to engage more actively in online forums than those with other cancer types [50,51].

Our study confirms that these spaces are relevant for sharing knowledge about extended AET, reflecting on interactions with HCPs, and exchanging advice and coping strategies—consistent with previous research [52]. The importance of online communities in sharing information and improving the emotional well-being of patients with breast cancer has been demonstrated [53,54]. These platforms provide effective means of identifying factors that influence patients’ adherence to AET [55]. A qualitative evidence synthesis further highlighted how informal support, both within online cancer communities and from fellow patients, can help women self-manage side effects and maintain adherence [26].

This underscores the significance of engaging with e-patients [52], who can both generate medical information for one another and comprehend complex information, sharing it effectively [56]. However, seeking information from other patients and forums also carries the risk of misunderstanding the clinical rationale for extending AET, as demonstrated in our study, or coming across inaccurate information about the treatment [57] and how to manage side effects.

Greater attention from HCPs to the role of online forums is warranted as these platforms help identify patients’ needs and concerns [35], including the questions and uncertainties women face when considering extended AET. Our findings suggest that a positive relationship with HCPs, based on trust in clinical advice, effective communication, and ongoing support, can encourage women to accept and continue extended treatment, especially when they are actively involved in decision-making [25]. In the context of treatment extension, tailored communication tools at key stages of the care pathway may facilitate more effective conversations between patients and HCPs about medication and side effects to monitor [25]. This is particularly important for side effects linked with extended AET, including the risks of endometrial cancer, thromboembolic events, and osteoporosis. Patient decision aids have proven to support shared decision-making and improve outcomes in breast cancer treatment, including AET [58-60].

Our study found that decision-making around extended AET may evolve over time, as shown in research on both 5-year [61,62] and 10-year regimens [39]. Concerns about extended AET can arise at any point in the treatment journey, causing uncertainty and reassessment of treatment decisions [57]. It is therefore crucial for clinicians to reengage with shared decision-making principles, using key points along the care pathway to review patients’ views. This would enable a reassessment of whether the potential benefits of extended treatment outweigh side effects by, for example, focusing on absolute rather than relative risk reduction and considering the impact on quality of life during treatment [55]. With the growing number of women undergoing extended AET, greater involvement of GPs and other trained HCPs (gynecologists, psychologists, and physiotherapists), as recommended by health authorities [29,30], and improved coordination between primary care (including pharmacists) and cancer centers could enhance treatment adherence and support comprehensive follow-up throughout the care pathway. It would also help tailor care to women’s personal circumstances and life plans (eg, desire for pregnancy) [63] and may reassure those for whom continuation of AET beyond 5 years is not medically necessary. Finally, it may also help address the emotional ambivalence experienced by some women at the end of extended AET and support a smoother transition out of treatment.

### Limitations and Strengths

This qualitative netnographic study has several limitations. First, the representativeness of women posting on breast cancer–related discussions is uncertain. Our findings are based on self-reported perceptions shared in an online environment by internet users, which may not fully reflect broader experiences of all women undergoing extended AET. Second, women who use online forums to express negative sentiments, including those related to treatment side effects, could be overrepresented as patients experiencing difficulties may be more likely to seek support in such communities as noted in the literature [64] and by some forum users themselves through their posts. Finally, the analysis was limited to data from 5 online forums, representing a limitation in terms of scope and generalizability of the findings.

Despite the limitations, the diversity of women’s profiles, the richness of the data, and the consistency of emerging themes support the robustness of the results and their relevance for informing recommendations around extended AET. A key strength of this study lies in a use of a variety of online discussion forums, with differing levels of activity and interaction styles. Some women engage in high-traffic, generalist forums where topics may extend beyond breast cancer, while others seem to favor close-knit communities focused on the disease or use these platforms primarily to seek factual medical information. The tone and content of forums range from informal personal narratives and emotional support to more formal, technical discussions, especially on platforms moderated by health care professionals. Posts also vary in length from brief comments to more detailed contributions. Although these differences do not permit definitive conclusions, it is noteworthy that a consistent set of themes was identified across platforms.

Given its *netnographic* nature and the researcher’s nonparticipant stance, this study does not interfere with forum interactions, thereby preserving spontaneity and authenticity [41]. Such interactions are less susceptible to social desirability bias than those in individual interviews, group discussions, or traditional ethnographic settings. Another key strength lies in longitudinal follow-up of some women across several years and different stages of their care trajectories. This provides insights into how treatment decisions evolve over time, in response to changing personal circumstances, updates in clinical guidelines, and, occasionally, interactions with other peers potentially influencing perspectives. Such dynamics are more challenging to capture through data collection methods where participants typically express themselves within a specific context and at a single point in time.

### Conclusions

Our study demonstrates that online forums are a valuable resource for understanding how women navigate extended AET, providing new insights into their proactive information-seeking from both medical and nonexpert sources. Understanding and acceptance of extended therapy were influenced by the timing and manner of information delivery. Decision-making patterns—ranging from acceptance to hesitation or refusal—may evolve throughout the treatment journey. These findings underscore the importance of providing clear, accessible information and personalized communication tools at the prescription and at key stages of the care pathway to support informed choices, foster adherence, and preserve quality of life. With the increasing number of women in long-term treatment pathways, strengthening coordination between primary care and hospital services, including follow-up by trained GPs and other HCPs, in collaboration with cancer centers, may help ensure continuity of care and comprehensive support.

This analysis of online forums will inform a follow-up study aimed at optimizing the communication about extended AET by integrating patients’ and HCPs’ perspectives and exploring the lived experience of long-term treatment.

## Supplementary material

10.2196/82016 Checklist 1 SRQR checklist.

## Abbreviations

AET: adjuvant endocrine therapy
AI: aromatase inhibitor
GP: general practitioner
HCP: health care provider
SRQR: Standards for Reporting Qualitative Research
TAM: tamoxifen
