# Supplementary material for: Women’s Insights on Extended Adjuvant Endocrine Therapy for Breast Cancer: Qualitative Online Forums Study
Source: J Med Internet Res. 2025 Dec 9;27:e82016. doi: 10.2196/82016 (PMC12688053; doi:10.2196/82016)
Supplement: Checklist 1 [file jmir-v27-e82016-s001.pdf]

Standards for Reporting Qualitative Research (SRQR)*

http://www.equator-network.org/reporting-guidelines/srqr/

Page/line no(s).

Title and abstract

**Title** - Concise description of the nature and topic of the study Identifying the study as qualitative or indicating the approach (e.g., ethnography, grounded theory) or data collection methods (e.g., interview, focus group) is recommended	Title page p.1
**Abstract** - Summary of key elements of the study using the abstract format of the intended publication; typically includes background, purpose, methods, results, and conclusions	Abstract page.1

Introduction

**Problem formulation** - Description and significance of the problem/phenomenon studied; review of relevant theory and empirical work; problem statement	Introduction p.2
**Purpose or research question** - Purpose of the study and specific objectives or questions	Introduction p.3

Methods

**Qualitative approach and research paradigm** - Qualitative approach (e.g., ethnography, grounded theory, case study, phenomenology, narrative research) and guiding theory if appropriate; identifying the research paradigm (e.g., postpositivist, constructivist/ interpretivist) is also recommended; rationale**	Described in Study Design and Data Analysis p 3 and p.4
**Researcher characteristics and reflexivity** - Researchers' characteristics that may influence the research, including personal attributes, qualifications/experience, relationship with participants, assumptions, and/or presuppositions; potential or actual interaction between researchers' characteristics and the research questions, approach, methods, results, and/or transferability	This has not been included as the study involved no interactions with forum users (non-participant approach).
**Context** - Setting/site and salient contextual factors; rationale**	Described in Study Design p.3 and 4 and Figure 1. (p.6)
**Sampling strategy** - How and why research participants, documents, or events were selected; criteria for deciding when no further sampling was necessary (e.g., sampling saturation); rationale**	Described in Data Collection p.4 and in Figure 2 (p.7)
**Ethical issues pertaining to human subjects** - Documentation of approval by an appropriate ethics review board and participant consent, or explanation for lack thereof; other confidentiality and data security issues	Described in Ethical Considerations p.5

**Data collection methods** - Types of data collected; details of data collection procedures including (as appropriate) start and stop dates of data collection and analysis, iterative process, triangulation of sources/methods, and modification of procedures in response to evolving study findings; rationale**	Described in Data Collection p.4 and 5 and Figure 2 (p.7)
**Data collection instruments and technologies** - Description of instruments (e.g., interview guides, questionnaires) and devices (e.g., audio recorders) used for data collection; if/how the instrument(s) changed over the course of the study	Described in Data Collection p.5
**Units of study** - Number and relevant characteristics of participants, documents, or events included in the study; level of participation (could be reported in results)	Described in Data Collection p.4 and 5 and Figure 2 (p.7)
**Data processing** - Methods for processing data prior to and during analysis, including transcription, data entry, data management and security, verification of data integrity, data coding, and anonymization/de-identification of excerpts	Described in Data Collection and Ethical Considerations p.5
**Data analysis** - Process by which inferences, themes, etc., were identified and developed, including the researchers involved in data analysis; usually references a specific paradigm or approach; rationale**	Described in Data Analysis p.5
**Techniques to enhance trustworthiness** - Techniques to enhance trustworthiness and credibility of data analysis (e.g., member checking, audit trail, triangulation); rationale**	Described in Data Analysis (use of Braun and Clarke's six-phase framework and multiple coders) p.5

Results/findings

**Synthesis and interpretation** - Main findings (e.g., interpretations, inferences, and themes); might include development of a theory or model, or integration with prior research or theory	Described in Results p.8 to 19 and Figure 3 (p.8)
**Links to empirical data** - Evidence (e.g., quotes, field notes, text excerpts, photographs) to substantiate analytic findings	Described in Results p.8 to 19, Figure 4 (p.13) and Figure 5 (p.16)

Discussion

**Integration with prior work, implications, transferability, and contribution(s) to the field -** Short summary of main findings; explanation of how findings and conclusions connect to, support, elaborate on, or challenge conclusions of earlier scholarship; discussion of scope of application/generalizability; identification of unique contribution(s) to scholarship in a discipline or field	Described in Discussion (p 19 to 22)
**Limitations** - Trustworthiness and limitations of findings	Limitations and Strengths section p.21

Other

**Conflicts of interest** - Potential sources of influence or perceived influence on study conduct and conclusions; how these were managed	Described in Conflicts of Interest p. 22
**Funding** - Sources of funding and other support; role of funders in data collection, interpretation, and reporting	Described in Funding Statement p.22

*The authors created the SRQR by searching the literature to identify guidelines, reporting standards, and critical appraisal criteria for qualitative research; reviewing the reference lists of retrieved sources; and contacting experts to gain feedback. The SRQR aims to improve the transparency of all aspects of qualitative research by providing clear standards for reporting qualitative research.

**The rationale should briefly discuss the justification for choosing that theory, approach, method, or technique rather than other options available, the assumptions and limitations implicit in those choices, and how those choices influence study conclusions and transferability. As appropriate, the rationale for several items might be discussed together.

Reference:

1. Bray F, Laversanne M, Sung H, Ferlay J, Siegel RL, Soerjomataram I, et al. Global cancer statistics 2022: GLOBOCAN estimates of incidence and mortality worldwide for 36 cancers in 185 countries. CA Cancer J Clin. 2024;74(3):229–63.
2. Santé publique France. Cancer du sein [Internet]. [cited 2025 Oct 7]. Available from: https://www.santepubliquefrance.fr/maladies-et-traumatismes/cancers/cancer-du-sein
3. Institut National du Cancer. Les cancers du sein [Internet]. 2025 [cited 2025 Oct 7]. Available from: https://www.cancer.fr/professionnels-de-sante/statistiques-et-chiffres-sur-les-cancers/epidemiologie-des-cancers/cancer-du-sein
4. Burstein HJ, Temin S, Anderson H, Buchholz TA, Davidson NE, Gelmon KE, et al. Adjuvant endocrine therapy for women with hormone receptor–positive breast cancer: American Society of Clinical Oncology Clinical Practice Guideline Focused Update. J Clin Oncol. 2014 Jul 20;32(21):2255–69
